# Supplementary figures and images for: Ameliorative Effects of Peptides from the Oyster (Crassostrea hongkongensis) Protein Hydrolysates against UVB-Induced Skin Photodamage in Mice
Source: Mar Drugs. 2020 May 31;18(6):288. doi: 10.3390/md18060288 (PMC7344810; doi:10.3390/md18060288)

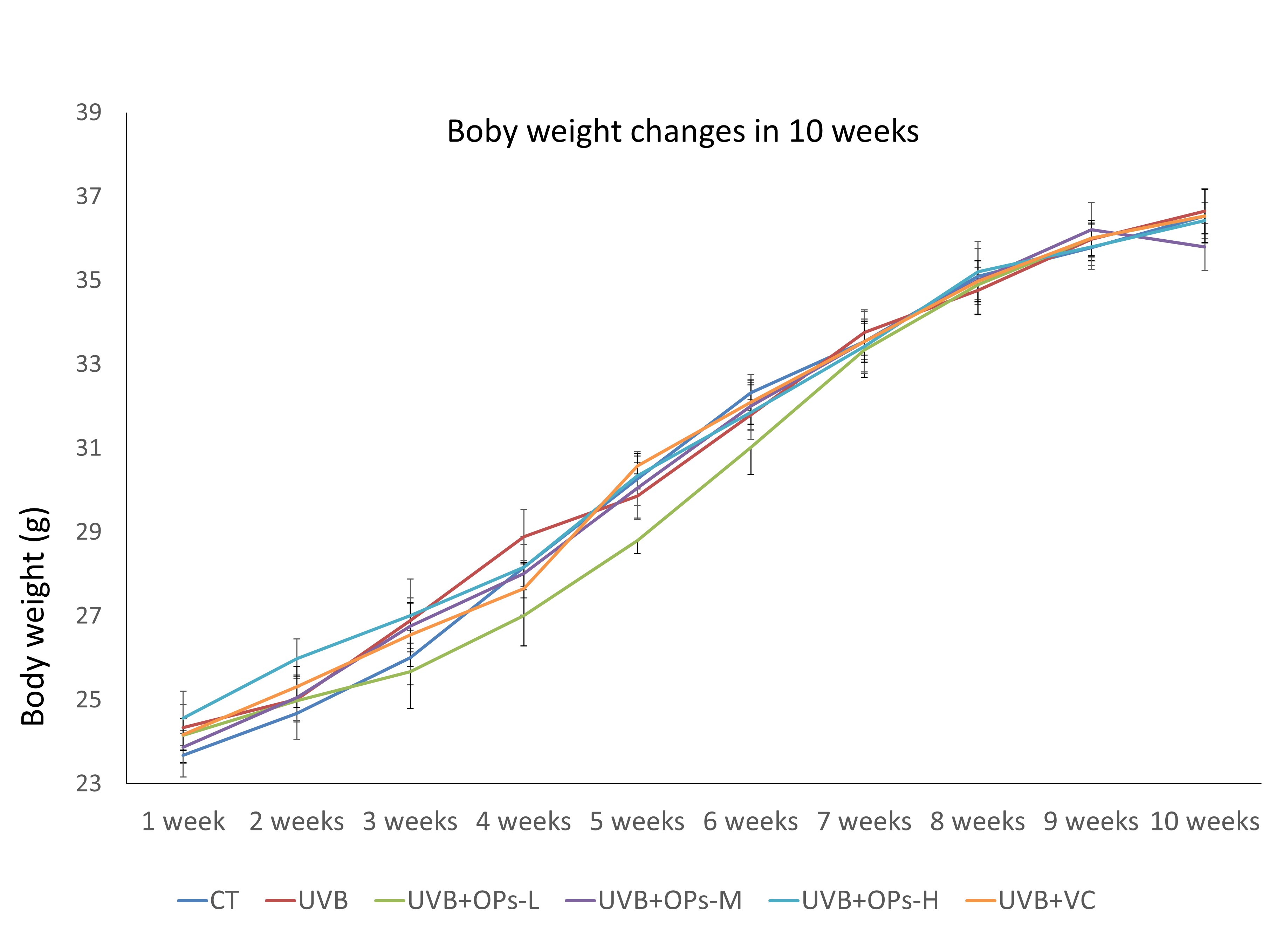

Supplement: Supplementary file 1 [file marinedrugs-18-00288-s001.zip › marinedrugs-796931-supplementary.jpg]
